# Whole-Genome Sequencing and Genomic Variant Analysis of Kazakh Individuals

**DOI:** 10.3389/fgene.2022.902804

**Published:** 2022-07-11

**Authors:** Ulykbek Kairov, Askhat Molkenov, Aigul Sharip, Saule Rakhimova, Madina Seidualy, Arang Rhie, Ulan Kozhamkulov, Maxat Zhabagin, Jong-Il Kim, Joseph H. Lee, Joseph D. Terwilliger, Jeong-Sun Seo, Zhaxybay Zhumadilov, Ainur Akilzhanova

**Affiliations:** ^1^ Laboratory of Bioinformatics and Systems Biology, Center for Life Sciences, National Laboratory Astana, Nazarbayev University, Nur-Sultan, Kazakhstan; ^2^ Laboratory of Genomic and Personalized Medicine, National Laboratory Astana, Nazarbayev University, Nur-Sultan, Kazakhstan; ^3^ Ilchun Genomic Medicine Institute, Seoul National University, Seoul, Korea; ^4^ Sergievsky Center, Departments of Neurology and Epidemiology, Taub Institute, Columbia University, New York City, NY, United States; ^5^ Departments of Genetics and Development and Psychiatry, Sergievsky Center, Columbia University, New York City, NY, United States; ^6^ School of Medicine, Nazarbayev University, Nur-Sultan, Kazakhstan

**Keywords:** Kazakh whole genomes, next-generation sequencing, genome analysis, human genetics, Kazakhstan, whole-genome sequence (WGS), whole-genome sequence analysis

## Abstract

Kazakhstan, the ninth-largest country in the world, is located along the Great Silk Road and connects Europe with Asia. Historically, its territory has been inhabited by nomadic tribes, and modern-day Kazakhstan is a multiethnic country with a dominant Kazakh population. We sequenced and analyzed the genomes of five ethnic Kazakhs at high coverage using the Illumina HiSeq2000 next-generation sequencing platform. The five Kazakhs yielded a total number of base pairs ranging from 87,308,581,400 to 107,526,741,301. On average, 99.06% were properly mapped. Based on the Het/Hom and Ti/Tv ratios, the quality of the genomic data ranged from 1.35 to 1.49 and from 2.07 to 2.08, respectively. Genetic variants were identified and annotated. Functional analysis of the genetic variants identified several variants that were associated with higher risks of metabolic and neurogenerative diseases. The present study showed high levels of genetic admixture of Kazakhs that were comparable to those of other Central Asians. These whole-genome sequence data of healthy Kazakhs could contribute significantly to biomedical studies of common diseases as their findings could allow better insight into the genotype–phenotype relations at the population level.

## 1 Introduction

Kazakhstan is the ninth-largest country in the world situated at the intersection of Europe and Asia. Historically, the territory of Kazakhstan has been inhabited by nomadic tribes, and at the present time, ethnic Kazakhs represent the majority in this multiethnic country.

Advances in next-generation sequencing (NGS) technologies allow sequencing of the entire genomes with a reasonable price, permitting a study of genetic variants in populations of interest. With the implementation of various efficient methods for the analysis of whole-genome sequencing (WGS), more comprehensive characterization of the genome is now possible. Prior to NGS advances, the knowledge of genetic variants relied primarily on the microarray data. However, shortage of predesigned microarray probes limited the discovery of novel variants (Wong et al., 2014).

Large-scale genomic studies can now address the important issue of the evolutionary history of human populations and comparative genomics (Xing et al., 2010). Unlike the advances in next-generation technologies, the collection of human DNA samples can be slow and unevenly distributed across different populations. Therefore, combined human genetic diversity datasets—the Human Genome Diversity Project (HGDP), HapMap, and 1000G projects—represent only a small subset of the global populations (Xing et al., 2010). Heyer et al. have provided detailed analysis of mitochondrial and Y-chromosome markers that highlighted the differences between different populations in Central Asia that are consistent with the anthropological data (Heyer at al., 2009). Unfortunately, the Kazakh genome data are limited in the aforementioned human genome databases. Only in 2020, Seidualy et al. published the results from genetic variant and admixture analysis using the WGS data of one healthy Kazakh female (Seidualy et al., 2020). However, Narasimhan et al. did report detailed analyses of hundreds of ancient individuals from the Central and South Asian regions, shedding light on genetic exchanges in Eurasia (Narasimhan et al., 2019).

Our study aims to perform the WGS of five healthy Kazakhs to provide insight into the genetic structure and diversity of Kazakhs in Kazakhstan. Specifically, we report WGS analysis of ethnic Kazakhs of both sexes using 30-fold coverage (26x–33x) generated by the Illumina HiSeq2000 NGS platform. The results of genomic analysis provide a valuable contribution to a better understanding of the genetic diversity and the landscape of the Central Asia region. Furthermore, sharing of these WGS data with the scientific community can serve as a valuable resource for comparative population studies and for biomedical studies for investigating disease associations.

## 2 Materials and Methods

### 2.1 Ethical Consideration, Sample Information, and DNA Extraction

The study protocol has been approved by the Institutional Review Board of the Center for Life Sciences IREC, Nazarbayev University (protocol #3, 4/04/2012). Study participants have agreed to share their genome data for the current and future studies and agreed to release their data to the public databases.

### 2.2 DNA Extraction, DNA Library Construction, and Whole-Genome Sequencing

Genоmic DNА was extracted from pеripheral bloоd using a Qiagen QI Amp mini kit. The concentration and quality of the isolated DNA were quantified using a NanoDrop spectrophotometer (Thermo Fisher Scientific, United States) and Qubit Fluorimeter (Thermo Fisher Scientific, United States). One μg of genomic DNA was used for paired-end DNA library preparation using a Illumina TruSeq DNA Preparation kit following the manufacturer’s recommendations (Illumina, United States). DNA libraries have been evaluated *via* Bioanalyzer 2,100 (Agilent Technologies, United States). DNA fragments were hybridized to the flow cell surface using the HiSeq paired-end cluster kit and later were amplified for the formation of clusters using Illumina cBot. The samples were sequenced using the Illumina HiSeq2000 NGS platform.

### 2.3 Bioinformatics Analysis

#### 2.3.1 Raw Data Preprocessing

Raw data files obtained from Illumina sequencing platforms in binary base call (bcl) format were converted to the fastq file format using the bcl2fastq v.2.20 tool. The quality of the generated sequences has been evaluated using FastQC v.0.11.7 (Andrews, 2010).

#### 2.3.2 Mapping of Sequencing Reads

Reads were aligned to the human reference genome (NCBI GRCh37, hg19) and reference mitochondrial DNA rCRS (NC_012,920) using Burrows–Wheeler Aligner v.0.7.12 (Li and Durbin, 2010) with default options and paired-end mode. Alignments corresponding to specific samples were combined to a single BAM file, and duplicates were marked using Picard tools v.1.130.

#### 2.3.3 Identification of Genomic Variants

Non-duplicate reads have been selected for downstream analysis, and the obtained BAM files were adjusted using base quality score recalibration (BQSR) and variant quality score recalibration (VQSR) procedure with default parameters. BQSR is a data preprocessing step that identifies systematic errors generated by a sequencing machine. VQSR is a complex filtering procedure that helps remove artifacts using machine learning techniques (Augoff et al., 2014). Genome Analysis Toolkit (GATK) v.3.7 and haplotype caller procedure have been used for genomic variant calling (Mckenna et al., 2010).

#### 2.3.4 Genomic Variants Analysis and Functional Annotation

Identified genomic variants were further annotated using ANNOVAR v. 2016Feb01 (Wang et al., 2010). The functional impact of the SNPs was then evaluated using SIFT (Ng and Henikoff, 2003) and PolyPhen (Adzhubei et al., 2010). Nonsynonymous SNPs were considered damaging if SIFT yielded a score ≤0.05 and PolyPhen-2 yielded a HVAR score ≥0.95. For gene-based annotation, three annotation databases (hg19_ALL.sites. 2015_08. txt; hg19-1000g2015_all; and dbSNP v.138/150) have been used. WebGestalt has been used for functional enrichment analysis of identified genetic variants (Wang et al., 2017).

#### 2.3.5 Principal Component Analysis and Admixture

Datasets. We used three datasets—namely, the Human Genome Diversity Project (HGDP), genotype dataset from Jorde lab, and 1000 Genomes Project dataset—for the comparative population analysis of Kazakh samples in relation to worldwide populations (Cavalli-Sforza, 2005; Xing et al., 2010; The 1000 Genomes Project Consortium, 2012). The Human Genome Diversity Project (HGDP) and Jorde lab dataset are the large-scale studies of human genome diversity. These datasets represent 64 different populations. The 1000 Genomes Project covers 2,500 individuals from 20 different populations worldwide. Principal Component Analysis. GCTA (Genome-wide Complex Trait Analysis) software was used to visualize the relationships between individuals from these datasets and assess the population structure. Prior to admixture analysis across different populations, PLINK v.1.07 (Purcell et al., 2007) was used to prune SNPs that are in high linkage disequilibrium. Subsequently, 113,290 SNPs and 129,777 SNPs were used for analysis. To convert VCF files to PLINK format files and compute Fixation Index (Fst), VCFTOOLS v.0.1.12b was used. Admixture Analysis. We performed the admixture analysis as implemented in ADMIXTURE v.1.23 by setting the number of ancestral population (k) to a range of 5–10 when comparing the membership of each Kazakh genome to dominant population groups (Alexander et al., 2009; Xing et al., 2010).

#### 2.3.6 Maternal and Paternal Ancestry Analysis

Every Kazakh individual was appointed to a unique mitochondrial haplogroup based on the whole sequences of mitochondrial DNA. Mitochondrial genomic variants have been identified using SamTools v.1.2 (Li et al., 2009). Then, haplogrep v.2 has been used for mitochondrial haplogroup identification and visualization (Kloss-Brandstatter et al., 2011). Y-chromosome haplogroups were manually determined for four Kazakh males in our group using ISOOG phylogenetic tree information and Y-chromosome genomic variants were identified. In addition, Yleaf kit v.2.1 (Thermo Fisher Scientific, United States) has been used for the identification and validation of Y-STR markers (Ralf et al., 2018).

## 3 Results

### 3.1 Whole-Genome Sequencing and Mapping Results

A total of five healthy Kazakh individuals, including four male and one female sample, from Kazakhstan were recruited, and their samples were sequenced with Illumina HiSeq2000 for achieving 30-fold coverage (Table 1 , Supplementary Table S1). For the alignment and mapping of each sequence generated, the hg19 reference genome was used. For the five Kazakh individuals, the total number of sequenced base pairs varies from 87,308,581,400 to 107,526,741,301, and on average, 99.06% were properly mapped. Sequencing quality was high as measured by the ratio of heterozygous SNVs to homozygous SNVs (Het/Hom) and transition/transversion (Ts/Tv) ranged from 1.35 to 1.49 and from 2.07 to 2.08, respectively. See Supplementary Table S2 for additional information on sequencing quality.

**TABLE 1:** Alignment statistics of whole-genome sequencing of Kazakh individuals.

Parameter	KAZ_WG2	KAZ_WG4	KAZ_WG5	KAZ_WG6	KAZ_WG7
In total reads	1,064,621,201	987,933,678	899,102,501	864,441,400	927,234,150
Duplicates	118,659,323	442,049,149	202,996,427	141,226,504	131,307,919
Mapped	1,060,168,691	976,604,161	889,481,111	855,464,242	917,897,457
Mapped (%)	99.58%	98.85%	98.93%	98.96%	98.99%
Singletons	2,363,888	8,184,471	7,569,803	6,525,956	7,505,472
Singletons (%)	0.22%	0.83%	0.84%	0.75%	0.81%
Throughput (bp)	107,526,741,301	99,781,301,478	90,809,352,601	87,308,581,400	93,650,649,150
Human Genome Fold Coverage (mapped)	33.10	30.49	27.77	26.71	28.66

### 3.2 Genomic Variants and Functional Annotation

Genetic variants were identified using the Genome Analysis Toolkit (GATK, version 3.7) and haplotype caller. The annotation of genetic variants was performed by SIFT, PolyPhen2, SNPedia, and ClinVar using ANNOVAR. The number of identified SNVs, insertions, and deletions for each individual are represented in Table 2.

**TABLE 2:** Total genetic variants identified in five Kazakh samples.

Sample	SNV	Novel SNP	MNVs	Novel MNVs	Deletions	Novel deletions	Insertions	Novel insertions
KAZ_WG2	3,158,814	14,898	209,294	29,841	312,682	15,856	312,420	33,077
KAZ_WG4	3,035,717	13,059	193,632	26,850	297,412	14,149	293,352	27,914
KAZ_WG5	3,110,974	14,122	202,611	28,370	306,392	15,069	305,205	30,820
KAZ_WG6	3,141,190	14,385	202,819	28,390	304,443	14,383	306,471	30,764
KAZ_WG7	3,131,384	15,011	204,417	28,801	14,163	28,813	308,449	32,003
AVERAGE	3,115,615.8	14,295	202,554.6	28,450.4	247,018.4	17,654	305,179.4	30,915.6

We identified a total of 15,578,079 SNVs and 2,760,989 indels (1,235,092 deletions and 1,525,897 insertions) in the five sequenced individuals. We identified novel variants that were not previously catalogued in the single-nucleotide polymorphism database dbSNP (avsnp138 and avsnp150). There are on average 247,018 deletions and 305,179 insertions among Kazakh individuals.

ANNOVAR was used to categorize SNPs into groups based on their genomic location and functional annotation (Supplementary Figures S1, S2). The frequency distribution of variants based on genome location shows that the majority of genetic variants were detected in intergenic (50.58%) and intronic (40.72%) regions, as expected (Supplementary Figure S1). The pie-chart of functional distribution (func.refGene) of all the variants and novel variants (separately) from five Kazakh individuals diagram illustrates that the intergenic (54.25%) and intronic (36.67%) variants were called with the highest frequency (Supplementary Figure S2).

We analyzed the length and number of indels based on their genomic locations. Indel sizes of variants were extracted from the VCF files, and their average number is shown in Supplementary Figure (Supplementary Figure S3). This figure illustrates log10 values of the count of genetic variants. Therefore, SNP variant number is displayed when indel size is 0. The number of mapped deletions was higher than that of insertions, and the length of deletions was greater than that of insertions.

Our study found 19,555 nonsynonymous somatic SNPs (nsSNPs) in all the five individuals (Supplementary Table S9). Out of a common 19,555 nsSNPs, 1,141 were homozygous nonreference (Supplementary Table S10) and among them 604 nsSNPs with a read depth higher than 5 were revealed as the private variants (Supplementary Table S11). Homozygous nonreference 1,141 nsSNPs were further scrutinized for over-representation analysis by WebGestalt tool. The results of analysis determined a significant enrichment of genes in several KEGG pathways (*p* < 0.05), such as olfactory transduction, complement and coagulation cascades, ATP-bind cassette (ABC) transporter, and ECM–receptor interaction (Supplementary Table S3). Only olfactory transduction remained statistically significant after calibrating for multiple testing with the False Discovery Rate (FDR) lower than 0.05.

The density of the identified nsSNPs on genomic regions is demonstrated on Supplementary Figure S4. Chromosomes 1 and 11 had a larger number of nsSNPs, more than 300 nsSNPs per chromosome (Supplementary Figure S4). Among these SNPs, 34 nsSNPs were predicted to be damaging to the protein product by SIFT, PolyPhen, and Provean (Supplementary Table S4, Supplementary Figure S5). Chromosomes 11 and 17 had the highest number of genomic variants which were predicted to be damaging. A number of olfactory genes and zinc finger protein family members were commonly found to be damaging.

### 3.3 Y-Chromosome and mtDNA Haplogroup Analysis

We identified the mitochondrial variants and assessed the mitochondrial haplogroups using HaploGrep (Kloss-Brandstatter et al., 2011). The samples belong to haplogroups H7a1, Z3c, J1b2, F1b1b, and T2b34. Haplogroup H7 was identified mostly in the European population, whereas other haplogroups were principally present in Asia (Wong et al., 2014).

We then analyzed the Y-chromosome haplogroups and haplotypes of Kazakh male individuals using Y-chromosome genetic variants and 17 STR-region identification approaches. Among the four Kazakh male samples examined, different haplogroups of the Y-chromosome were identified (Supplementary Table S5). Two Kazakh samples were allocated to haplogroup R1, which were mostly identified in Central Asia. Another two have been designated to haplogroups N1a and O2a. Y-STR region profiles of analyzed samples have been accessible in Supplementary Table S6.

### 3.4 Admixture and Principal Component Analysis

#### 3.4.1 Principal Component Analysis

**FIGURE 1:**
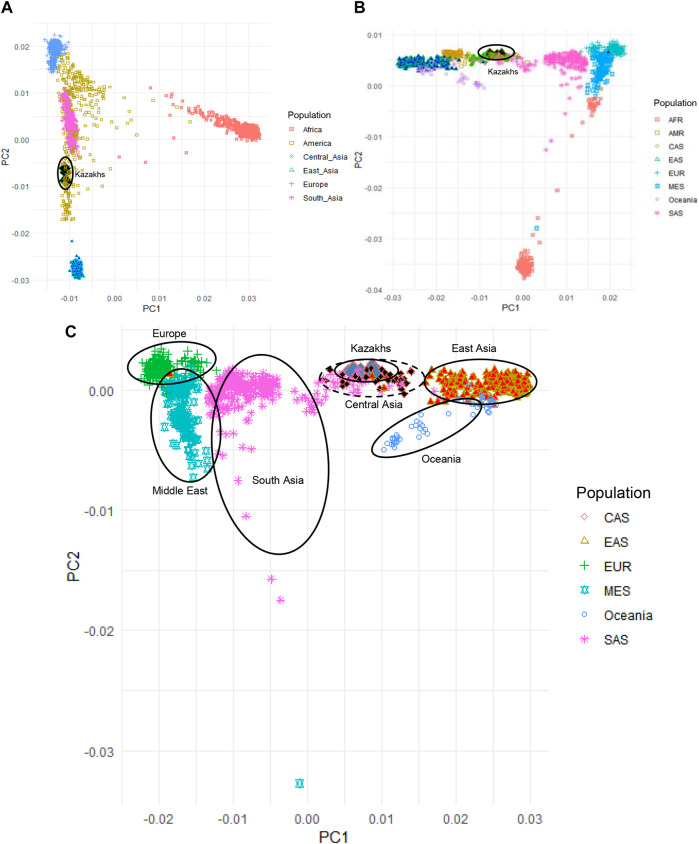
Principal component analysis (PCA) of Kazakh samples along with samples from worldwide populations from 1000G, HGDP, and Jorde genomic projects. **(A)** PCA plot of Kazakh samples and 1000 G project; **(B)** PCA plot of Kazakh samples with the HGDP project; **(C)** PCA plot of Kazakh samples with Eurasian populations from the HGDP and Jorde genomic projects. Kazakh samples are highlighted in blue rhombus. AFR-Africa, AMR-America, CAS—Central Asia, EAS-East Asia, EUR-Europe, MES—Middle East, and SAS-South Asia.

Principal component analysis (PCA) was applied to evaluate the population structure of the Kazakh samples with representatives of worldwide populations from 1000G, HGDP, and Jorde genomic project for understanding the ancestral origins of the Kazakh population (Figure 1). PCA analysis of the Kazakh samples with populations from 1000G databases (The 1000 Genomes Project Consortium, 2012) is demonstrated in Figure 1A. We have repeated the PCA analysis with populations from the HGDP project to validate our findings (Figure 1B). PCA analysis of the Kazakh individuals with several Central Asian and Eurasian populations selected from the HGDP and Jorde genomic projects is represented in Figure 1C and shows the position of the Kazakh samples on the genetic map across Eurasia.

#### 3.4.2 ADMIXTURE Analysis

**FIGURE 2:**
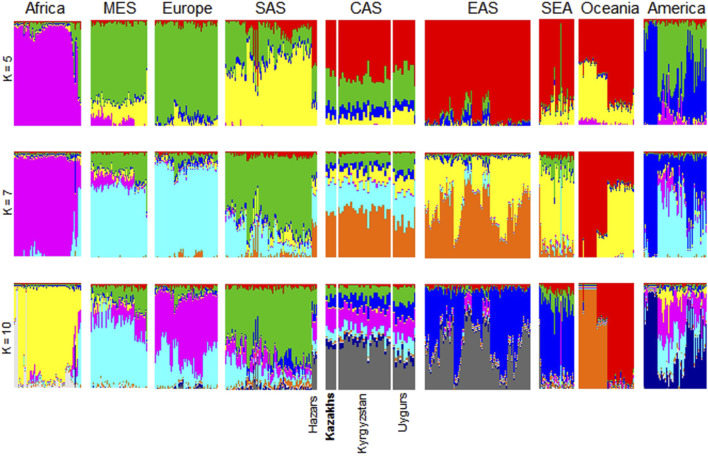
ADMIXTURE plots for Kazakhs assuming a different number of ancestral populations. These figures were generated using genomic data from 3,805 individuals collected from the 1000G, HGDP, and Jorde genomic projects. ADMIXTURE analysis assumed ancestral populations (k) to be 5, 7, or 10. The samples were clustered according to their geographical location to represent their genetic structure. Particularly, Central Asians (i.e., Hazars, Kazakhs, Kyrgyzstan, and Uygurs) had comparable levels of genetic admixture when varying Ks were assumed. AFR-Africa, AMR-America, CAS—Central Asia, EAS-East Asia, EUR-Europe, MES—Middle East, and SAS-South Asia.

ADMIXTURE conducts the unsupervised clustering of a great number of samples and at the same time, each individual can be represented as a combination of clusters (Alexander et al., 2009). To evaluate the population admixture for each sample in the worldwide to dominant population groups, we performed admixture analysis of 3,805 samples collected from 1000G, HGDP, and Jorde genomic projects (Cavalli-Sforza, 2005; The 1000 Genomes Project Consortium, 2012) together with Kazakh samples (Figure 2). The number of ancestral populations was fixed to range from five to ten. Kazakh individuals are genetically diverse population at the whole-genome level and show similar ancestral patterns with populations from Central Asia. As shown in Figures 1, 2, ethnic Kazakhs comprised European and Asian admixture, as expected.

### 3.5 Disease Association and Pathways

Variants from all the five healthy (i.e., non-diseased) individuals were further checked for the functional analyses of variants with SNPEDIA databases (Cariaso and Lennon, 2012). Following the annotation effort *via* SNPEDIA, we have extracted only those genetic variants that were common in all the five samples with a selection magnitude ≥1, a recommended subjective ratio by SNPEDIA. A total of 32 variants were found to have a negative impact on disease (Supplementary Table S7). Among our samples, we found three genetic variants (rs9300039, rs11037909 in EXT2 and rs3740878 in EXT2) that potentially increase the risk for type 2 diabetes. We also identified genetic variants associated with metabolic syndrome, hypertension (rs1805762 in M6PR), and increased body weight (rs5746059 in TNFRSF1B). Moreover, several genetic variants were found to increase risk for neurodegenerative diseases, such as Alzheimer’s disease (rs4938369 in BACE1) or schizophrenia (rs6932590 in TRNAV27). We note that it is difficult to assess the value of the findings since the identified genetic variants from this small set of non-diseased individuals is inconclusive since 1) they may later develop the disease or 2) the risk variants identified from different populations may have little impact on ethnic Kazakhs due to differing genetic and environmental backgrounds. We will address these possibilities in our future studies when we have an adequate number of clinically diagnosed cases vs. controls.

In addition, we applied the Combined Annotation Dependent Depletion (CADD, C score ≥20) and FATHMM-MLK (Damaged) databases for prediction of the potential functional impact of SNVs. A total of 189 genetic variants affecting 164 different genes met these filtering criteria. We then analyzed overrepresented diseases in these 164 genes by curating information from the DisGeNET database by the utilization of WebGestalt. Supplementary Table S8 shows the list of overrepresented diseases with *p*-value <0.05 and corresponding FDR values. There are three significantly affected changes with FDR <0.05, such as muscular dystrophy and other dystrophic changes.

### 3.6 Data Availability

Sequencing data have been uploaded to the NCBI SRA read archive under accession number PRJNA374772 (Kairov et al., 2021). The VCF file that contains all the genomic variants described in this study is available upon request.

## 4 Discussion

Advances in NGS technologies have allowed sequencing of the entire genome for potentially large-scale genomic projects. Once such data are generated, and they can be used on a wide range of topics from comparative genomics to other health studies that predict or estimate genetic risks or even to treatment studies. Most existing human genome databases include a limited number of populations and a limited number of individuals within, focusing on European populations. Consequently, no Kazakh genomes are represented in most databases. In this study, we present the comprehensive analysis results of WGS data of ethnic Kazakhs generated using the NGS platform. The WGS data were obtained at high coverage (29.3X on average) for four men and one woman from Kazakhstan. The alignment of the obtained genomic sequences on reference genome hg19 has shown that an average of 99.06% was mapped. Quality assessment of data based on the ratios of heterozygous SNVs to homozygous SNVs (Het/Hom) and transition/transversion (Ts/Tv) ratios demonstrated that our whole-genome sequences were acceptable to the standard practice in the field. This study provides a useful application in biomedicine and makes valuable contributions to our understanding of the genetic landscape and the diversity of Central Asian populations.

Genetic variant analysis of five sequenced Kazakh individuals has identified a total of 15,578,079 SNVs and 2,760,989 indels with further annotations. Novel genetic variants were identified for each individual (Table 2). The SNPs have been categorized based on genomic location and annotation (Supplementary Figures S1, S2). Functional analysis of all the common nonsynonymous somatic SNPs (nsSNPs) among five Kazakhs revealed several pathways with significant enrichment of genes (Supplementary Table S3). Further analysis of these pathways has demonstrated that only olfactory transduction remained significant after multiple testing corrections with false discovery rate (FDR). This is consistent with an earlier study by Gudbjartsson et al. that showed significant enrichment of olfactory genes in a large-scale WGS of the Icelandics (Gudbjartsson et al., 2015). Genomic mapping of identified genetic variants across human chromosomes have shown that chromosomes 1 and 11 have larger number of nsSNPs (Supplementary Figure S4). Altogether, 34 nsSNPs that are predicted to be damaging to the protein product were identified using SIFT, PolyPhen, and Provean prediction methods. Specifically, a number of olfactory gene families (mostly on chromosome 11) and zinc finger proteins were identified.

We have performed PCA, admixture, and identification of haplogroups based on Y-chromosome and mitochondrial DNA. Based on the PCA that compared ethnic Kazakhs to worldwide populations from 1000 Genomes Project, HGDP, and Jorge genome project (Figure 1), the first principal component (PC1) distinguished African populations from all other populations, which indicates that the first PC divided populations according to their genetic heterogeneity. The second principal component (PC2) mostly divided the population based on their longitudinal location and heterogeneity (Figure 1A). When compared with the populations from the HGDP project, repeated PCA analysis yielded a similar distribution of samples and consistent with the findings from 1000G project, where PC1 distinguished populations based on the longitudinal distribution from west to east (PC1) and genetic diversity of population (PC2; See Figure 1B). In addition, we have performed PCA analysis on Kazakhs with several Central Asian and other Eurasian samples selected from the HGDP and Jorde genomic projects to further characterize the position of the Kazakh samples in the genetic maps of human populations in Eurasia (Figure 1C). PC1 and PC2 mainly reflected the geographic distribution of the populations, with the majority of genetic variations explained by their locations (Xing et al., 2010). The comparison of populations genetically supports the fact that the Kazakh population is located in the middle of the European and East Asian populations.

Taking the abovementioned results one step further, the assignment of mitochondrial haplogroups of Kazakh individuals demonstrated that four out of five Kazakh samples were set to haplogroups that were prevalent in Asia, and only haplogroup H7a1 was mostly found in Europe. Analysis of Y-chromosome haplogroups of Kazakh men identified four different haplogroups, two of which were assigned to R1 haplogroups, mostly found in Central Asia, and another two were defined as haplogroup N1a and O2a. The diversity of the Kazakh Y-chromosome haplogroups has been reported previously (Tarlykov et al., 2013; Balanovsky et al., 2015; Zhabagin et al., 2017), and a large number of variants were reported. A comparison of the examined male haplotypes with the national database reveals similar variants within five mutational steps for three haplotypes (WG2, WG4, and WG5) (Zhabagin et al., 2019).

Admixture analysis of the population SNP data provided a degree of admixture of populations for each sample. It showed that ethnic Kazakhs are genetically admixed and share similar ancestral patterns to other Central Asians (Figure 2). We observed that ethnic Kazakhs were consistently admixed between the populations of Europe and Asia, such as Hazaars, Kyrgyzstan, and Uygurs. The majority of Hazaars, a historical nomadic Turkish tribe, now reside in Pakistan. Although Hazaran samples were gathered from Pakistan, they are genetically similar to Central Asians than to Pakistanis as shown in several studies (Rosenberg et al., 2002; Li et al., 2008). Therefore, Hazarans were grouped together with Uygur and Kyrgyz populations as Central Asians (Hodoglugil and Mahley, 2012). Throughout history, the ethnic Kazakhs have been a nomadic group that has migrated in different regions of Central Asia, leading to a high degree of admixture with local populations (Hodoglugil and Mahley, 2012). Historical migration of the ethnic Kazakhs reflects the genetic structure of the current day ethnic Kazakhs. This study is helpful in identifying the place of the Kazakh population relative to the worldwide populations.

WGSs of ethnic Kazakhs also provide a valuable contribution to biomedical research and further improvement of diagnostics by finding disease associations for various genetic variants. Functional analysis of genetic variants using SNPEDIA databases has identified 32 variants that may have negative effects on various diseases. Many genetic variants were associated with symptoms of metabolic syndrome, such as hypertension (rs1805762), type 2 diabetes (rs9300039, rs11037909, and rs3740878), and increased body weight (rs5746059). Higher risk for neurodegenerative diseases has been linked with several genetic variants (rs4938369 and rs6932590). We have revealed that muscular dystrophy was overrepresented among analyzed ethnic Kazakhs.

In conclusion, this WGS study further characterized the genetic structure and diversity of five unrelated ethnic Kazakhs in comparison to world populations. We showed high genetic admixture of Kazakh genomes at the autosomal level and similar complex genetic heterogeneity of Central Asians. These whole-genome sequences of healthy Kazakh individuals provide invaluable resources for further studies of modern human origin and evolution, causal variants for Kazakh characteristic disease/traits, and personal medicine. The genomic data of a larger number of Kazakh individuals will help answer these questions in the contexts of population research and personalized medicine.

## Data Availability

The datasets presented in this study can be found in online repositories. The names of the repository/repositories and accession number(s) can be found below: https://www.ncbi.nlm.nih.gov/, PRJNA374772.
